# THE INCIDENCE AND IMPACT OF POLYPHARMACY IN AL RESIDENTS

**DOI:** 10.1093/geroni/igz038.878

**Published:** 2019-11-08

**Authors:** Elizabeth Galik

**Affiliations:** University of Maryland School of Nursing, Baltimore, Maryland, United States

## Abstract

The purpose of this study was to describe polypharmacy in AL settings. We hypothesized that: (1) age, gender, race, setting, multimorbidity and cognitive status would influence polypharmacy; and (2) polypharmacy would be associated with falls, emergency room visits and hospitalizations. This was a descriptive study using data from the first cohort of the FFC-AL-EIT Study. A total of 242 participants from 26 AL settings were included. Participants had a mean age of 86.86 (SD=7.0), the majority were women 179(74%) and white (N=232, 96%) with 5 (SD=2) comorbidities. The mean number of drugs was 7 (SD=3.56) and 51% were exposed to polypharmacy, 24% fell at least once, 9% were sent to the hospital and 13% to the emergency room. Neither hypothesis was supported. Continued research is needed to explore the factors that influence polypharmacy and to identify if there are negative outcomes associated with polypharmacy in this population.

